# Breastfeeding and Respiratory Infections in the First 6 Months of Life: A Case Control Study

**DOI:** 10.3389/fped.2019.00152

**Published:** 2019-04-24

**Authors:** Elisabetta Pandolfi, Francesco Gesualdo, Caterina Rizzo, Emanuela Carloni, Alberto Villani, Carlo Concato, Giulia Linardos, Luisa Russo, Beatrice Ferretti, Ilaria Campagna, Alberto Tozzi

**Affiliations:** ^1^Multifactorial Disease and Complex Phenotype Research Area, Bambino Gesù Children's Hospital (IRCCS), Rome, Italy; ^2^Department of Pediatrics, Bambino Gesù Children's Hospital (IRCCS), Rome, Italy; ^3^Virology Unit, Laboratory Department, Bambino Gesù Children's Hospital (IRCCS), Rome, Italy

**Keywords:** respiratory infection, breastfeeding, prevention, viral infection, pediatrics, breast milk

## Abstract

**Background:** Viral respiratory tract infections (VRI) are a major reason for hospitalization in children younger than 5 years. A case control study was conducted to investigate the potential role of breastfeeding in protecting children <1 year of age from VRI.

**Methods:** Patients admitted for a respiratory tract infections routinely underwent a nasopharyngeal aspirate, which was tested with an RT-PCR for 14 respiratory viruses. Hospitalized infants positive for viruses were enrolled as cases; healthy controls were enrolled among patients admitted for ultrasound hip screening. The effect of breastfeeding on pertussis was investigated through multivariable analysis.

**Results:** We enrolled a total of 496 patients: 238 cases and 258 healthy controls. Among cases, eighty-six patients (36.1%) had a rinovirus, 78 (32.8%) an RSV, 22 (9.2%) an adenovirus, and 37 (15.5%) a coinfections with multiple viruses. The number of households was significantly higher in cases (mean in cases 4.5; mean 3.7 in controls, *p* < 0.001) and the proportion of infants having siblings (79% in cases vs. 43% in controls, *p* < 0.001). Proportion of smoking mothers was higher in cases than in controls (21.4 vs. 10.1%, *p* = 0.001). Among cases 44.5% were exclusively breastfed at symptoms onset vs. 48.8% of healthy controls. According to the multivariable analysis, being exclusively breastfed at symptom onset was associated with a higher risk of viral respiratory infection (3.7; 95% CI 1.64–8.41), however a longer breastfeeding duration was protective (OR 0.98; 95% CI 0.97–0.99). Also having at least one sibling was associated to a higher risk (OR 3.6; 95% CI 2.14–5.92) as well as having a smoking mother (OR 2.6; 95% CI 1.33–4.89).

**Conclusions:** Breastfeeding remains a mainstay of prevention for numerous diseases and its protective role increases with duration. However, being breastfed when mothers carry a respiratory infection may increase the risk of transmission, acting as a proxy for closer contacts. In future studies, potential confounding variables as pattern of contacts with other individuals, should be taken into account.

## Background

Respiratory tract infections are a leading cause of morbidity in children.

Studies conducted in industrialized countries report a prevalence of respiratory tract infections ranging from 3.4 to 32.1% in the first year of life (1–4). Respiratory tract infections are also a major reason for hospitalization in children younger than 5 years (5–7).

Different studies have explored and confirmed the role of clinical and socioeconomic risk factors for respiratory tract infections, including birth weight, gestational age, socioeconomic status, ethnicity, number of siblings, day care attendance, and parental smoking (4, 8, 9). Breastfeeding is included among the protective factors for respiratory infections in infants. The protective role of breastfeeding against respiratory infections has been repeatedly demonstrated for children living in developing countries (10–12). Although breastfeeding is described as protective also in industrialized countries, different study designs, definitions (e.g., for infant feeding and kind of infection), timing of evaluation of exposure (feeding), and outcome (infection), have been used in studies performed this setting.

Most studies confirm a protective role of breastfeeding against respiratory infections in the long term, as the outcomes are often measured after 6 months of age, or even at 1, 2, or 6 years (13), showing a persistent protective effect even after breastfeeding has been stopped.

In fact, protection seems to be time dependent: in a large cohort of infants in the UK, those who were breastfed for <4 months had a higher risk of hospitalization for infectious diseases in the first year of life compared with those who were breastfed for more than 4 months (14). In addition, infants who were breastfed for 4–6 months showed a higher risk of both pneumonia and recurrent otitis media compared to those who were breastfed for 6 months or longer (15–17).

Fewer are the studies analyzing the protective role of breastfeeding in the first 3 months of life. While Duijts et al. report a protective effect of breastfeeding in children exclusively breastfed until 4 months of age compared to never breastfed infants (1) other studies report a weaker protection for children younger than 3 months (18) or no protection at all (19).

We report here the results of a case-control study exploring the association of breastfeeding with viral respiratory infections in a metropolitan area, in children younger than 12 months of age.

## Methods

### Study Design and Setting

We conducted a case-control study in one large metropolitan pediatric hospitals, located in Rome, Italy, between June 2012 and February 2018. Bambino Gesù Children's Hospital is the largest pediatric research hospital in Europe. It accounts for nearly 600 beds and admits several patients from outside the Lazio Region, especially those with chronic and severe diseases. It performs every year a total of over 1 million and 690 thousand pediatric clinical encounters.

The study was supported by the ECDC (European Center for Disease Control) within the Pertinent project (Pertussis in Infants European Network), a European hospital-based network dedicated to measuring pertussis burden in infants and to studying pertussis vaccine effectiveness.

The objective of the present study was to analyze the role of breastfeeding as a protective factor for viral respiratory infections in children younger than 6 months of age. Data on the effect of breastfeeding on pertussis were reported in a recently published paper (20).

Patients admitted for a respiratory tract infection routinely underwent a nasopharyngeal aspirate, which was tested with a real time polymerase chain reaction (RT-PCR) for 14 respiratory viruses (listed in the next paragraph).

Patients <6 months of age with a positive RT-PCR for one of these viruses were enrolled as cases.

A group of healthy controls aged < 6 months were systematically enrolled among healthy infants admitted as outpatients in the same hospital for hip ultrasound screening, on Tuesdays and Thursdays, in the same period during which cases were recruited. Infants with a previous hospitalization were excluded.

### Nasopharyngeal Aspirate Collection and RT-PCR for Viruses

Nasopharyngeal aspirates were performed and processed using a specific panel detecting the following viruses: RSV, influenza virus A and B, human coronavirus OC43, 229E, NL-63, and HUK1, adenovirus, hRV, parainfluenza virus 1–3, human metapneumovirus-hMPV, and human bocavirus-hBoV.

Samples of nasopharyngeal aspirate were collected within 24 h from hospital admission and processed immediately, or stored at −70°C until performing the test.

Nucleic acids were extracted from a 200 μl sample of nasopharyngeal aspirates and purified, using the EZ1 Virus Mini Kit v. 2.0 on the EZ1 Advanced XL platform (Qiagen, GmbH, Hilden, Germany). Nucleic acid extracts were eluted into 90 μl of buffer and processed immediately.

### Data Collection

For each enrolled patient (cases and controls), the following data were recorded: socio demographic data, gestational age, kind of delivery, birth weight, parents' level of education and employment, kind of feeding at symptom onset (exclusive breastfeeding, partial breastfeeding, artificial feeding), number of households, number of smokers in the family.

Data were collected through a questionnaire administered to parents of patients at enrolment, after signing an informed consent.

Epidemiological data were recorded in an electronic database (Microsoft Access).

### Statistical Analysis

In Italy, the prevalence of exclusive breastfeeding at 3 months has been estimated to be nearly 60% (21). Considering this estimate, we calculated that a sample of 490 patients was sufficient to show an odds ratio of 0.6 for exclusively breastfed infants vs. infants with partial breastfeeding or artificial feeding, with a power of 80 and a 95% confidence level.

Proportions were compared using the Chi-square test or the Fisher exact test. Differences between means were studied through the Student's *T*-test. A *P*-value < 0.05 was considered statistically significant.

As the aim of our study was to analyse the effect of exclusive breastfeeding on the risk of respiratory infections, we decided to include in the same group artificial feeding + partial breastfeeding at symptom onset or at enrolment. A multivariable logistic regression analysis was performed in order to study the effect of exclusive breastfeeding (exclusive vs. partial breastfeeding or artificial feeding, at symptom onset for cases, or at enrolment for controls) and its duration (days) on the occurrence of respiratory tract infections, adjusted for the following variables: age (days), sex (male vs. female), ethnicity (caucasian vs. non caucasian), gestational age at birth (weeks), birth weight (kg), kind of delivery (vaginal vs. cesarean), parents' employment, parents' level of education (university degree vs. lower), parents' smoking habits, number of households, having at least 1 sibling.

Multicollinearity between the independent variables was assessed by studying the correlation matrix and examining the tolerance and the variance inflation factor (VIF).

Stata 13 was used for statistical analysis.

## Ethical Approval

The study was approved by the Bambino Gesù Children's Hospital Ethical Committee (protocol RF-2010-2317709).

## Results

### Population

Socio-demographic characteristics of the study population are shown in Table 1. We enrolled a total of 496 patients: 238 cases and 258 healthy controls. Healthy controls were older than cases (mean age of controls 2.38 months, mean age of cases 1.99 months, *p* < 0.001). The proportion of premature babies was significantly higher in cases than in controls (21 vs. 7%, *p* < 0.001). The proportion of mothers and fathers with a university degree was higher in controls (for mothers 39.5 vs. 28.6% respectively, *p* = 0.010; for fathers, 31.4 vs. 21.9% respectively, *p* = 0.017). The number of households was higher in cases (mean 4.5) than in controls (mean 3.7, *p* < 0.001), and so was the proportion of infants having siblings (79% in cases vs. 43% in controls, *p* < 0.001). Proportion of smoking mothers was higher in cases than controls (21.4 vs. 10.1%, *p* = 0.001).

**Table 1:** Socio-demographic characteristics of patients enrolled.

	**Respiratory infections (cases) (*N* = 238)**	**Healthy (controls) (*N* = 258)**	**Total (*N* = 496)**	***p*-value**
Male (*n*, %)	112 (47.1)	129 (50.0)	241 (48.6)	0.513
Age in months (mean, sd)	2.0 (1.1)	2.4 (0.8)	2.2 (1.0)	< 0.001
Caucasian (*n*, %)	216 (90.8)	240 (93.0)	456 (91.9)	0.354
Gestational age, weeks (mean, sd)	37.7 (2.6)	38.9 (1.9)	38.4 (2.3)	< 0.001
Premature birth (*n*, %)	50 (21.0)	18 (7.0)	68 (13.7)	< 0.001
Birth weight in kg (mean, sd)	3.0 (0.6)	3.2 (0.5)	3.11 (0.58)	< 0.001
Caesarean birth (*n*, %)	117 (49.2)	107 (41.5)	224 (45.2)	0.086
Employed mother (*n*, %)	138 (58.0)	158 (61.2)	296 (59.7)	0.460
Employed father (*n*, %)	221 (92.9)	238 (93.0)	459 (92.9)	0.961
Mother with university degree (*n*, %)	68 (28.6)	102 (39.5)	170 (34.3)	0.010
Father with university degree (*n*, %)	52 (21.9)	80 (31.4)	132 (26.8)	0.017
Households (mean, sd)	4.5 (1.8)	3.7 (0.9)	4.1 (1.5)	< 0.001
One or more brothers (*n*, %)	188 (79.0)	111 (43.0)	299 (60.3)	< 0.001
Smoker mother (*n*, %)	50 (21.4)	26 (10.1)	76 (15.5)	0.001
Smoker father (*n*, %)	80 (34.2)	83 (32.2)	163 (33.1)	0.635

Among children with an acute respiratory tract infection, 19.3% of mothers had respiratory symptoms.

### Viral Characteristics

Of 238 patients with respiratory infections, eighty-six patients (36.1%) had a rhinovirus infection, 78 (32.8%) a respiratory syncytial virus, 22 (9.2%) adenovirus, and 37 (15.5%) had coinfections with multiple viruses, such as Coronavirus, Metapneumovirus, Influenza A and B, Parainfluenza virus.

The median duration of hospital admission was 6 days with no differences by type of virus. Among those with respiratory infections, a total of 74 patients (31.1%) had complications during hospital admission, and nearly a half of these (49.4%) had a RSV infection.

### Breastfeeding

Table 2 describes the kind of infant feeding at symptom onset.

**Table 2:** Kind of breastfeeding at symptoms onset in the study population.

	**Respiratory infections (*N* = 238)**	**Healthy (*N* = 258)**	**Total (*N* = 496)**	***p***
Patients with exclusive breastfeeding at symptom onset (*n*, %)	106 (44.5)	126 (48.8)	232 (46.8)	0.626
Days of exclusive breastfeeding prior to enrollment (mean, sd)	35.8 (42.97)	41.6 (36.97)	38.8 (40.02)	0.110
Never breastfed (*n*, %)	85 (35.7)	72 (27.9)	157 (31.7)	0.062

A proportion of 46.8% of enrolled patients were exclusively breastfed at enrollment. Among patients with respiratory tract infections, 44.5% were exclusively breastfed at symptoms onset (Table 2) while 48.8% of patients were exclusively breastfed at enrolment among healthy controls. The mean duration of exclusive breastfeeding was 35.8 days in cases and 41.6 days in controls.

Among cases, 19% of children had a mother with respiratory symptoms while breastfeeding. No differences were observed in breastfeeding duration between cases having a mother with symptoms and those without.

In addition, we found that nearly 80% of household contacts of cases had respiratory symptoms at their enrollment.

The mean length of hospital stay was 7.6 days in children exclusively breastfed compared to 12.5 days in those with partial breastfeeding or artificial feeding, but this difference was not statistically significant.

Among cases, mothers with a university degree were 32.1% among those who were exclusively breastfeeding at symptom onset and 25.8% among those providing partial breastfeeding or artificial feeding. As for controls, mothers with a university degree were 47.6% among those who were exclusively breastfeeding at symptom onset and 31.8% among those providing partial breastfeeding or artificial feeding.

### Multivariable Analysis

According to the multivariable analysis, having at least one sibling was associated to a higher risk of viral respiratory infection (OR 3.6; 95% CI 2.14–5.92) as well as having a smoking mother (OR 2.6; 95% CI 1.33–4.89). Being exclusively breastfed at symptom onset was associated with a higher risk of viral respiratory infection (3.7; 95% CI 1.64–8.41) but protection increased with breastfeeding duration (OR 0.98; 95% CI 0.97–0.99) (Table 3).

**Table 3:** Factors associated to the risk of having an acute viral respiratory infection according to a logistic regression model.

	**OR**	**95%CI**	***p***
Age (days)	0.99	0.98–1.00	0.043
Male	0.85	0.55–1.30	0.456
Caucasian	0.77	0.35–1.73	0.534
Gestational age, weeks	0.84	0.73–0.96	0.012
Birth weight, grams	0.71	0.43–1.17	0.174
Vaginal birth	1.30	0.83–2.04	0.250
Mother with university degree	0.69	0.40–1.20	0.190
Father with university degree	0.67	0.39–1.17	0.160
Employed mother	1.12	0.69–1.80	0.654
Employed father	1.32	0.56–3.15	0.527
Exclusive breastfeeding	3.7	1.64–8.41	0.002
Breastfeeding duration, days	0.98	0.97–0.99	0.001
Number of households	1.16	0.93–1.44	0.189
One or more siblings	3.56	2.14–5.92	< 0.001
Smoking mother	2.55	1.33–4.89	0.005
Smoking father	0.76	0.47–1.23	0.264

## Discussion

Breastfeeding is a mainstay for the prevention of infectious diseases. The protective effect of prolonged breastfeeding against infectious diseases in children living in developing countries has been well documented (22, 23). However, protection from respiratory infections through breastfeeding in developed countries has not been uniformly demonstrated, with major differences between methods in most studies (11, 12, 24–28).

With the present study, we confirm risk factors previously identified for respiratory infections in infants: low gestational age, young age, having one or more siblings (29, 30).

Moreover, in our study, we confirm an association between maternal smoking and the risk of having a viral respiratory infection (OR 2.55; 95% CI 1.33–4.89). As other authors reported, children of smoking mothers have an increased risk of severe RSV infection, morbidity, mortality and hospitalizations for respiratory infection and other infectious diseases (31), SIDS (32), wheezing and asthma (33–35).

The association of maternal smoking and respiratory conditions seems to be linked to the interference of nicotine on lung development (36).

Regarding breastfeeding, our results confirm the time-dependent effect of exclusive breastfeeding in the protection against VRI, which is actually in line with the majority of published studies (1, 18, 25). Nevertheless, at the same time, the multivariable analysis identified exclusive breastfeeding as a risk factor for VRI. Some of the previously published literature are in line with this result. Some authors showed that breastfeeding does not provide substantial protection against common infectious illnesses during the first year of life (24). Other studies concluded that a shorter period of breastfeeding might increase the risks of illness and physician visits for lower respiratory tract infections (29). Some authors reported that breastfed babies do not have fewer respiratory viral infections or illnesses, although they may experience less severe disease (28).

Evidence on the protective role of breastfeeding against infections of the gastrointestinal tract is more robust (37) compared to findings on the protection from respiratory infections. This has an immunological explanation. Breastmilk actually contains numerous protective factors such as immunoglobulins, lactoferrin, and lymphocytes, as well as other factors that may contribute to reduce infant mortality in developed countries (25). As a matter of fact, immunoglobulins ingested through breastfeeding confer a direct, timely protection against microorganisms localized in the gastrointestinal tract, which are directly bound by ingested breast milk IgA. On the other hand, in order to protect from infections localized in other body sites, ingested immunoglobulins should be absorbed through the intestinal mucosa and move to other areas through the bloodstream. Nevertheless, evidence shows that the process of intestinal IgA absorption is not effective (37, 38).

One of the factors that should be taken into account when evaluating the transmission of an infective agent is the pattern of contacts among individuals, which has been shown to affect the risk of viral infections in other studies (39, 40).

We recently published a study exploring the pattern of contacts within the households of infants younger than 6 months, through the use of Radio-Frequency Identification devices (41). We showed that families in which the baby is exclusively breastfed tend to have a more intense contact pattern compared to families in which the infant receives artificial of mixed feeding—not only the contacts of the infant with the mother are more frequent and of a longer duration, but also the contacts of the infant with the father. In other words, breastfeeding may represent a proxy for closer contacts of the infant with the mother and, possibly, with other household members. As contact patterns vary in different country settings (42), also the influence of breastfeeding on contact patterns may vary depending on geographic contexts.

In our study, among cases, nearly 80% of all household members had respiratory symptoms at enrollment and 19% of mothers had acute respiratory symptoms while breastfeeding, suggesting the possible association between mother's/household members' proximity and risk of infection. The increased risk due to increased proximity is higher in the first months of life, and is probably counterbalanced in older infants by the development of the immune system and by other long-term, immune-modulating effects of breast milk that still need to be elucidated.

This study has several potential limitations.

The first limitation concerns the study design: we could not determine parents' symptoms among controls and we were not able to measure the pattern of contacts within household members. A cohort study taking into account contact patterns in the households would be appropriate to confirm these observations, although the execution of a study with such characteristics would entail a complex organization and require high investments. Moreover, cases were younger than controls, but this potential confounding effect was adjusted through the multivariable analysis.

Finally, as our aim was to assess the effect of exclusive breastfeeding on the risk of respiratory infections, we decided to include children with partial breastfeeding and those with artificial feeding in the same subgroup. This is a typical issue in the design of studies on breastfeeding and health outcomes and might have biased in the resulting effect of breastfeeding on respiratory infections. Nevertheless, our retrospective study design did not allow to assess the specific dose of breastmilk received by the enrolled infants.

## Conclusion

Overall, we strongly support the choice of exclusive breastfeeding as the best possible kind of feeding for infants in the first months of life. Our results support the evidence that breastfeeding effect toward protection against VRI is dose dependent: the longer the duration of exclusive breastfeeding, the stronger the protection. In addition to, we suggest that, in future studies aimed at assessing the breastfeeding protective role for airborne diseases, potential confounding variables as pattern of contacts with other individuals, together with contact symptoms, have to be taken into account, to avoid bias in interpretation.

Moreover, taking into account the potentially higher probability of infection transmission in families with frequent and long contacts among household members, we support the adoption of recommendations issued by the Center for Disease Control and Prevention for the prevention of VRI transmission to infants. According to these recommendations, symptomatic mothers should thoroughly wash their hands with soap and water before touching the infant and cover their nose and mouth with a tissue when sneezing or coughing in close contact with the infant (43).

Exclusive breastfeeding should be promoted for the first six months of life, as WHO and CDC strongly recommend, because the risk of infant morbidity for viral acute respiratory infections is negatively associated with the duration of breastfeeding.

## Ethics Statement

This study was carried out in accordance with the recommendations of name of guidelines, name of committee with written informed consent from all subjects. All subjects gave written informed consent in accordance with the Declaration of Helsinki. The protocol was approved by the Bambino Gsù ethical committee.

## Author Contributions

All the authors have contributed significantly to the study, and have seen and approved the present correspondence. EP contributed to the conceptualization of the study, wrote, and reviewed the entire manuscript. FG contributed in writing the manuscript and to the oversight of the final revision. CR reviewed the manuscript and contributed to methodology section. EC provided the statistical analysis of the data and contributed to data curation. AV contributed to the revision of the manuscript. CC and GL provided microbiological data and contributed to write the results sections. LR, BF, and IC contributed to study design and revised the entire manuscript. AT contributed to the conceptualization of the study, wrote, and reviewed the entire manuscript.

### Conflict of Interest Statement

AT has received research grants for vaccine studies from Sanofi Pasteur MSD, Pfizer and Glaxo SmithKline. The remaining authors declare that the research was conducted in the absence of any commercial or financial relationships that could be construed as a potential conflict of interest.
